# The Impact of Estrogen Deficiency on Liver Metabolism: Implications for Hormone Replacement Therapy

**DOI:** 10.1210/endrev/bnaf018

**Published:** 2025-06-16

**Authors:** Jiawen Dong, Kaitlyn M J H Dennis, Radha Venkatakrishnan, Leanne Hodson, Jeremy W Tomlinson

**Affiliations:** Oxford Centre for Diabetes, Endocrinology and Metabolism, NIHR Oxford Biomedical Research Centre, University of Oxford, Churchill Hospital, Oxford OX3 7LJ, UK; Oxford Centre for Diabetes, Endocrinology and Metabolism, NIHR Oxford Biomedical Research Centre, University of Oxford, Churchill Hospital, Oxford OX3 7LJ, UK; Department of Gynaecology, Oxford University Hospitals NHS Foundation Trust, Oxford OX3 9DU, UK; Oxford Centre for Diabetes, Endocrinology and Metabolism, NIHR Oxford Biomedical Research Centre, University of Oxford, Churchill Hospital, Oxford OX3 7LJ, UK; Oxford Centre for Diabetes, Endocrinology and Metabolism, NIHR Oxford Biomedical Research Centre, University of Oxford, Churchill Hospital, Oxford OX3 7LJ, UK

**Keywords:** estrogen, hormone, replacement, MASLD, NAFLD, liver

## Abstract

Metabolic dysfunction-associated steatotic liver disease (MASLD; previously nonalcoholic fatty liver disease) is the most common chronic liver condition globally. It affects 1 in 3 individuals and is associated with increased liver and cardiovascular mortality. MASLD is a sexually dimorphic condition, and in women the prevalence and severity of MASLD rises significantly following menopause. Preclinical data shows that lack of estrogen promotes multisystem metabolic dysfunction that is characteristic of MASLD. This not only includes hepatic lipid accumulation, insulin resistance, and fibrosis but also extra-hepatic metabolic processes in adipose and skeletal muscle. There are currently no available MASLD treatments tailored to women. The uptake of estrogen-based menopausal hormone replacement therapy (HRT) has seen a dramatic increase in recent years. Despite the changing attitudes toward HRT and the strong evidence base implicating estrogen deficiency in the development of MASLD, the impact of HRT on MASLD in postmenopausal women is poorly studied. In this review, we discuss the burden of MASLD in women, the effect of estrogen deficiency on the processes that drive MASLD development and progression, and the potential sex-specific therapeutic strategies that may prevent or limit MASLD development after menopause.

## Essential Points

- The liver is an estrogen-responsive organ, and estrogen-deficient murine models display hepatic lipid accumulation, inflammation, and fibrosis.
- Estrogen deficiency leads to adverse systemic metabolic changes in skeletal muscle, adipose, and the gut, which further promote the MASLD phenotype.
- Despite the evidence for an increased risk of MASLD and MASLD fibrosis after menopause, no specific therapeutic strategies exist to address to prevent or reverse MASLD development at this stage of life.
- Menopausal HRT is a candidate treatment strategy for MASLD; however, existing studies have yielded conflicting results.
- Further research is required to clarify the effect of HRT on MASLD and provide insights on the impact of route of administration and the progesterone component of HRT.

Menopause is a turning point for the development of metabolic dysfunction-associated steatotic liver disease (MASLD) (previously termed nonalcoholic fatty liver disease). MASLD is the most common chronic liver disease, affecting 30% of people, and its prevalence is projected to rise further, fueled at least partly by the epidemic of diabetes and obesity (1). The prevalence of MASLD is lower in premenopausal women compared to age-matched men; however, after menopause, prevalence rates rise in women, ultimately being comparable to, or even higher than, men (2, 3). MASLD is a condition characterized by intrahepatic lipid accumulation, which can progress to inflammation [metabolic dysfunction-associated steatohepatitis (MASH)], fibrosis, and eventually cirrhosis (4). MASLD fibrosis is associated with increased liver-related morbidity and mortality but also an increased risk of cardiovascular disease and its associated mortality, the prevailing cause of death in patients with the disease (5). The clinical outcomes from MASLD are particularly poor in women, where MASLD mortality rates are rising faster in women than in men (6). Furthermore, MASLD is the leading indication for liver transplant in women (7). The cessation of ovarian estrogen production at menopause is strongly implicated in MASLD development (8). Preclinical studies have established the wide-ranging effects of estrogen on metabolic processes, and rodent models of estrogen deficiency have demonstrated many features of the multisystem metabolic dysregulation that drive MASLD development (9-11).

Although MASLD is a sexually dimorphic condition exacerbated by hormonal changes, women over the age of 50 years are currently underrepresented in clinical studies of liver disease (12). The high burden of disease and poor outcomes in women emphasize the need for the development of tailored treatment strategies for women. Despite the wealth of evidence that characterizes the metabolically unfavorable changes in hepatic pathways linked to estrogen deficiency (8, 13), the potential for estrogen-based hormone replacement therapy (HRT) to limit the development of MASLD has not been rigorously tested. It is not clear whether HRT mitigates or promotes the processes that drive MASLD, and this is particularly concerning as HRT is becoming a more prevalent treatment option. Within the United Kingdom, the number of HRT prescriptions per financial year has increased more than 4-fold from 2015 to 2024 (14).

This review outlines the impact of estrogen deficiency on the metabolic processes that contribute to MASLD development and progression. The prevalence of MASLD in postmenopausal women is described alongside other evidence linking estrogen deficiency in women with heightened MASLD risk. Using data from preclinical rodent and human studies, the mechanisms of hepatic estrogen signaling, the role of estrogen in regulating hepatic substrate utilization, and estrogen's anti-inflammatory and antifibrotic effects in the liver are discussed. Furthermore, we examine how estrogen deficiency disrupts extrahepatic metabolic processes and contributes to metabolic inflexibility within other tissues, including adipose tissue and skeletal muscle, and leads to gut microbial dysbiosis. Finally, we review the current clinical evidence on the impact of menopausal HRT on MASLD in menopausal women and discuss potential treatment strategies for MASLD following menopause.

## Hepatic Estrogen Signaling

Estrogen signaling is a highly regulated, tissue-specific process that depends on the local and circulating concentration of estrogen, the expression of estrogen receptors within the target tissue, and the presence of other cell-specific machinery. There are 4 major endogenous estrogens in humans: estrone (E1), 17β-estradiol (E2), estriol (E3), and estetrol (E4). E2 is the most potent estrogen and the predominant circulating estrogen in premenopausal women, where it is primarily produced by the ovarian follicle and functions as a circulating hormone to effect distant target tissues. In postmenopausal women, where ovarian E2 production has ceased, E1 becomes the predominant endogenous estrogen. However, E2 continues to be produced postmenopausally in extragonadal sites from the aromatization of androgens, where it acts locally in an autocrine or paracrine manner (15, 16). Circulating estrogens in postmenopausal women are thought to be derivates of estrogen metabolism in extragonadal sites, rather than bioactive molecules driving estrogen action in distant tissues (15). The effect of local postmenopausal production of E2 within the liver is likely to be relatively small compared to the effect of systemic E2 exposure in the premenopausal state (17).

**Figure 1.**
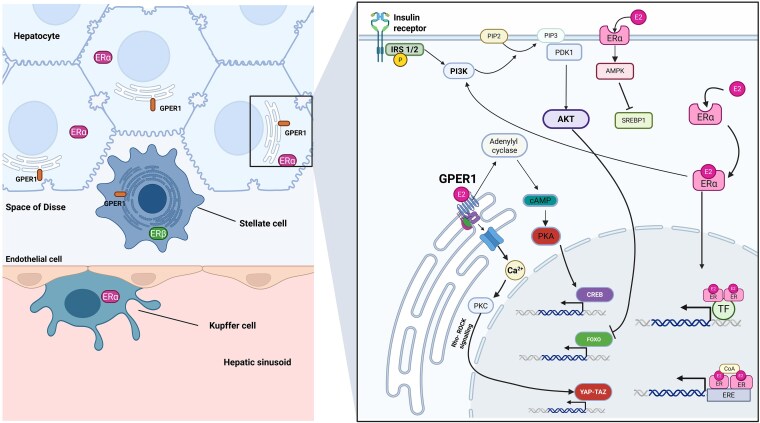
Estrogen signaling in the liver. Left panel: Cellular architecture of hepatic sinusoids and the expression of predominant estrogen receptors (ERs) within cell types. Sinusoids are lined by fenestrated endothelial cells and contain Kupffer cells (resident macrophages), which express ERα. The space of Disse (perisinusoidal space) between the sinusoid and the surrounding hepatocytes contains hepatic stellate cells, which express ERβ and G-protein coupled estrogen receptor 1 (GPER1). Hepatocytes wrap around the space of Disse and predominantly express ERα and GPER1. Right panel: Hepatocyte estrogen signaling via ERα and GPER1. GPER1 resides predominantly on intracellular membranes such as the endoplasmic reticulum and Golgi apparatus. Activation of GPER1 leads to multiple downstream signaling cascades, including the release of Ca^2+^ from intracellular stores to activate protein kinase C (PKC) and activation of Rho-rock signaling to regulate YAP-TAZ transcription factors, cAMP production to activate protein kinase (PKA), and subsequently the cAMP response element-binding protein (CREB) transcription factor. Upon activation, ERα dimerizes and translocates to the nucleus where it acts as a transcription factor by directly binding to estrogen response elements or interacting with coactivators (CoA) to regulate gene expression. Upon activation of membrane-bound ERα, the phosphorylation of sterol regulatory element-binding protein 1 (SREBP-1) by AMP-activated protein kinase (AMPK) is triggered. Phosphorylation of SREBP-1 prevents its cleavage and nuclear translocation. Insulin receptor tyrosine kinase activation in the liver leads to phosphorylation of insulin receptor substrate (IRS) 1 and 2, which leads to activation of 1-phosphatidylinositol 3-kinase (PI3K), catalyzing the conversion of phosphatidylinositol (4,5)-bisphosphate (PIP2) to phosphatidylinositol (3,4,5)-trisphosphate (PIP3). This activates 3-phosphoinositide-dependent protein kinase 1 (PDK1) and subsequently Akt. Akt inhibits the transcription factor Forkhead box protein O (FOXO) to regulate the transcription of gluconeogenic genes. Created in BioRender. Tomlinson, J. (2025) https://BioRender.com/r34p991.

Abbreviations: AMPK, AMP-activated protein kinase; CoA, coactivators; CREB, cAMP response element-binding protein; ER, estrogen receptor; ERE, estrogen response elements; FOXO, Forkhead box protein O; GPER1, G-protein coupled estrogen receptor 1; IRS, insulin receptor substrate; PDK1, 3-phosphoinositide-dependent protein kinase 1; PI3K, 1-phosphatidylinositol 3-kinase; PIK1, 3-phosphoinositide-dependent protein kinase 1; PIP2, phosphatidylinositol (4,5)-bisphosphate; PIP3, phosphatidylinositol (3,4,5)-trisphosphate; PKA, protein kinase A; PKC, protein kinase C; SREBP-1, sterol regulatory element-binding protein 1.

Three distinct estrogen receptors (ER) have been discovered: ERα, Erβ, and G-protein coupled estrogen receptor 1 (GPER1). ERα and ERβ are “classical” nuclear receptors, encoded by ESR1 on chromosome 6 and ESR2 on chromosome 14, respectively (18). Upon ligand activation, these nuclear ERs dimerize and translocate to the nucleus where they act as transcription factors by binding to estrogen response elements directly or interact with coactivators such as activator protein 1 or specificity protein 1, to regulate gene expression (19). A population of membrane-localized ERα has been described, and this portion of ERα mediates signal transduction via AMP–activated protein kinase (AMPK) to regulate cellular metabolic activity (20) (Fig. 1).

In rodents and humans, ERα is expressed in hepatocytes from both sexes where it plays an important role in regulating lipid and glucose metabolism (8, 21-23). In rodents, concentrations of nuclear ERα show age-dependent, but not sex-dependent, variations with higher nuclear concentrations observed in the perinatal and postpubertal phases of life (23). In female mice, hepatic ERα transcriptional activity oscillates in phase with the estrous cycle and appears to coordinate the synchronous oscillation of hepatic genes controlling lipid metabolism (10). The tight regulation of lipogenic genes by the estrous cycle is crucial for controlling lipid homeostasis. Lipogenic genes are nonoscillatory in ovariectomized and liver ERα knockout mice where greater liver lipid accumulation is observed in the livers (10). Liver-specific knockout of ERα in female rodents leads to increased lipid droplet deposition in the liver, increased hepatic inflammatory gene expression, and increased liver collagen deposition (24). There is no significant long-term change in liver ERα mRNA expression after ovariectomy. However, after a 12-week treatment with E2, liver ERα expression significantly increased in ovariectomized compared to sham-operated rats receiving the same treatment, suggesting that long-term estrogen replacement may lead to enhanced hepatic estrogen responsiveness, although this phenomenon is yet to be demonstrated in estrogen-deficient humans receiving estrogen replacement (25). ERα also appears to modulate the activity of liver resident macrophages, Kupffer cells, in response to hepatic injury (26).

In the rodent and human liver, the expression of ERβ is much lower in comparison to ERα (21). Unlike ERα knockout rodents, the hepatic metabolic phenotype of ERβ knockout rodents does not appear to be affected (27). However, functional ERβ is expressed in hepatic stellate cells (21, 28) where it appears to modulate fibrogenesis (29).

The GPER1 gene encodes a 7-transmembrane domain G-protein coupled receptor that is located mainly on intracellular membranes such as the endoplasmic reticulum (30). Activation of GPER1 leads to intracellular calcium release and activation of heterotrimeric G proteins to trigger various genomic and non-genomic pathways (Fig. 1) (30). GPER1 regulates a diverse range of metabolic and anti-inflammatory functions across wide-ranging tissues (30) and is expressed in rodent and human hepatocytes (11, 31). The role of GPER1 in hepatic metabolism has been elucidated in hepatocyte-specific GPER1 knockout female mice, which possess more pronounced high-fat diet induced insulin resistance, hepatic steatosis, hepatic inflammation, and fibrosis compared to GPER1-Flox control female mice (11). The impact of GPER1 activation on fibrogenesis may be mediated by the presence of GPER1 in hepatic stellate cells (HSCs) where it regulates the mechanical activation of HSCs (32).

## Epidemiology of MASLD and the Association Between Estrogen Deficiency and MASLD in Humans

The endocrine changes that characterize perimenopause usually start at approximately 45 years of age, and the median age of menopause in women from developed countries (defined by a duration of amenorrhea for 1 year) is between 50 and 52 years (33). Epidemiological studies on MASLD prevalence have consistently shown that patterns of disease prevalence in men and women across ages are distinct and that disease prevalence is higher in women compared to men after the age of 50 years (34). The prevalence of MASLD in men increases across adulthood before plateauing at midlife and falling at 50 to 60 years, whereas MASLD prevalence in women increases after 50 years, peaking at 60 to 69 and falling after 70 years (34). In premenopausal women, MASLD prevalence is lower than in men. This trend is overturned after the age of 50 years, where the prevalence of MASLD in women rises to reach a similar level to men of the same age (34). Interestingly, some studies report an even higher age-matched prevalence in women (35). The higher prevalence of MASLD after menopause is significant even after correcting for age and body mass index (BMI) (13, 36, 37). Furthermore, surgically induced menopause from oophorectomy is an independent risk factor for MASLD, and oophorectomy at a younger age is associated with a higher risk of MASLD (38). A recent meta-analysis reported an odds ratio (OR) for the association between menopause and MASLD of 2.19 [95% confidence interval (CI) 1.73-2.78] after adjusting for age and metabolic factors such as BMI and glycemic status (36). There is limited data on the association between menopausal stages and the prevalence of MASLD; however, if the risk of MASLD is linked with declining circulating E2 levels, MASLD risk would not be expected to increase in early perimenopause where E2 levels do not consistently fall and may even periodically rise above premenopausal levels (see Fig. 1). Consistent with this hypothesis, 1 cross-sectional study in middle-aged Korean women assessed MASLD prevalence during perimenopause and following transition to postmenopause and found that the prevalence of MASLD during early perimenopause stages was not significantly different from premenopausal stages (37). Compared to premenopausal stages, a higher prevalence of MASLD was observed in women in both late perimenopause or postmenopausal stages, where estrogen levels typically decline (37). Further reduction of E2 produced locally in the liver in postmenopausal women may further increase MASLD risk. Postmenopausal women with breast cancer using aromatase inhibitors, which reduce local conversion of androgens to E2, appear to have a higher risk of MASLD compared to healthy postmenopausal women, independent of BMI or diabetes status (39).

**Figure 2.**
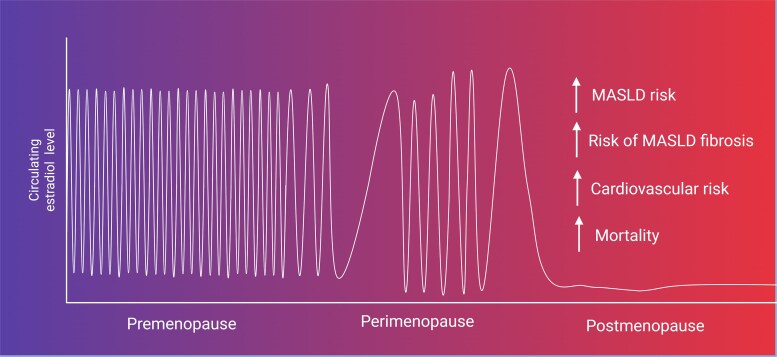
Risk of metabolic dysfunction-associated steatotic liver disease (MASLD) and changes in estrogen levels during life. During adulthood, circulating estradiol levels fluctuate during the menstrual cycle. Toward midlife, as ovarian reserve declines, the individual enters perimenopause. Cyclical circulating estradiol levels become erratic during perimenopause, and during early perimenopause women may experience prolonged high levels of serum estradiol intermittently. The reasons for this are complex and are related to a decline in ovarian inhibin production from declining ovarian follicle reserve. Inhibin suppresses pituitary release of follicle stimulating hormone (FSH), and falling inhibin levels permit a slight increase in serum FSH levels—this accelerates follicle maturation and may lead to a phenomenon of “luteal-out-of-phase” phenomenon and a surge of estradiol in the luteal phase (42). As the ovarian follicle reserve depletes further, women experience longer periods of amenorrhea, and it is not until late perimenopause that estradiol levels decline (43). In the postmenopausal state, when estradiol levels are low, the risk of MASLD and MASLD fibrosis is higher, which is accompanied by an increased risk of cardiovascular disease and mortality. Created in BioRender. Tomlinson, J. (2025) https://BioRender.com/wvl0ab5.

Abbreviations: MASLD, metabolic dysfunction-associated steatotic liver disease.

Biopsy studies have demonstrated that a longer duration of estrogen deficiency increases the risk of fibrosis in women with MASLD. Time from menopause (calculated by age at study enrollment—age at menopause) was significantly associated with fibrosis (adjusted cumulative OR for 5-year unit = 1.2, 95% CI 1.1-1.3, *P* = .002) (40). Premature menopause was associated with an increased risk of fibrosis after adjusting for multiple confounders, including BMI (adjusted cumulative OR 1.9, 95% CI 1.3-2.7, *P* = .001) (40). This association between menopause and increased risk of hepatic fibrosis has been validated in a nonobese Japanese cohort of women with biopsy-proven MASLD, where postmenopausal women had more severe fibrosis compared to the premenopausal women after adjusting for confounders (OR 2.173, *P* = .0266), further supporting the existence of a relationship is that independent of obesity (41). The relationship between circulating estrogen levels and MASLD risk is displayed in Fig. 2.

Another estrogen-deficient population that appears to have an increased risk of MASLD is people with Turner syndrome, who develop hypergonadotropic hypogonadism. A high prevalence of elevated liver enzymes (50%) (44), steatosis (65%), and fibrosis (39%) (45) is observed in women with Turner syndrome. Taken together, evidence from preclinical models and observational clinical data would all appear to endorse the conclusion that estrogen-deficient states increase the risk of MASLD.

## Genetic Polymorphisms Modify the Risk of MASLD Progression at Menopause

MASLD is a disease with a strong genetic component, and recent studies have shown that genetic variants interact with sex and menopausal status to modify the risk of MASLD progression. A biopsy-linked genetic analysis of 98 359 single nucleotide polymorphisms (SNPs) associated with metabolic pathways identified 25 SNPs that significantly interact with sex or menopausal status to affect the risk of MASLD fibrosis, of which 10 independent loci remained after linkage disequilibrium clumping to remove genomic correlation (46). Notably, *KCNIP4* rs12501548, *KLHL8* chr4:88356546, rs349715 (intergenic), and *NOTCH2* rs4659248 all demonstrated a detrimental effect on MASLD fibrosis stage in postmenopausal but not premenopausal women. The rs738409 C > G SNP of patatin-like phospholipase domain-containing 3 (*PNPLA3*) encoding the p.I148M variant, which accounts for the largest proportion of disease heritability, has a functional interaction with hepatic ERα that drives MASLD susceptibility (47). *PNPLA3* is highly expressed in hepatocytes and HSCs and encodes a protein that resides in lipid droplets with triglyceride hydrolase activity. The p.I148M substitution appears to abolish the hydrolase activity by restricting the substrate from reaching the active site of the enzyme (48). There is a multiplicative interaction between female sex and the PNPLA3 p.I148M polymorphism on hepatic steatosis, lobular inflammation, and fibrosis stage (47). An ERα-binding site is located within a PNPLA3 enhancer, and ERα activation in multilineage hepatic spheroids increases PNPLA3 expression, resulting in lipid accumulation and fibrogenesis (47). A clearer understanding of how genetic variants associated with MASLD interact with sex and reproductive status will allow for risk stratification of MASLD progression at the time of menopause. In addition, this would have the potential to identify new treatment targets and promote the development of personalized treatment strategies.

## Metabolic Effects of Estrogen Deficiency Within the Liver

### Hepatic Lipid Metabolism

Hepatic lipid content is determined by the balance between the processes of lipid influx and synthesis [de novo lipogenesis (DNL), fatty acid delivery, and esterification] vs disposal (fatty acid oxidation and lipid export from the liver). Important components of the cellular machinery controlling these processes are under transcriptional, translational, and posttranslational control by estrogen, and in vivo studies in humans have demonstrated sex-specific differences (49). Estrogen deficiency ultimately disrupts the balance of hepatic lipid influx/synthesis and disposal, leading to liver lipid accumulation and lipotoxicity (49).

DNL, the synthesis of palmitate from various substrates including sugars, involves a series of enzymatic reactions catalyzed by ATP-citrate lyase, acetyl-CoA carboxylase (ACC), and fatty acid synthase (20). Fatty acid synthase and ACC are transcriptionally regulated by E2 treatment in HepG2 cells (50, 51), and both ERα- and GPER1-knockout rodent models display upregulated hepatic expression of genes involved in lipid biosynthesis (27). ERα binding sites are enriched on promoters of genes involved in lipid synthesis and fatty acid metabolism (52), and 1 of these binding sites is located in the promoter of the transcription factor STAT3 (53). Following long-term treatment of leptin-deficient *ob/ob* mice with E2, levels of STAT3 and phosphorylated STAT3 on tyrosine 705 in the liver increase, downregulating the expression of lipogenic genes ATP-citrate lyase, and fatty acid synthase (53). E2 replacement in ovariectomized mice decreases hepatic expression of sterol regulatory element-binding protein 1c (SREBP-1c), a master transcriptional regulator of lipogenic genes in hepatocytes (54). ACC activity is also posttranslationally regulated by E2 (49). Plasma membrane-bound ERα activation appears to be key in AMPK-mediated phosphorylation of ACC in the liver, inactivating the enzyme (20). AMPK signaling decreases the transcription of lipogenic genes by phosphorylation of transcription factors SREBP1 and carbohydrate-responsive element binding protein, which control the expression of fatty acid biosynthetic genes (55, 56). Activation of hepatic GPER1 leads to cAMP release and AMPK-mediated signaling (11), and activation of membrane-bound ERα triggers the protein kinase A-liver kinase B-AMPK signaling pathway, increasing phosphorylation of ACC and SREBP1 (20). An in vivo study in humans using stable isotope tracers has shown that women (mean age 46 years, SD 6 years, menopausal status not specified) have a significantly lower contribution of DNL in very low density lipoprotein triglyceride (VLDL-TG) postprandially compared to age-matched men (57). Longitudinal studies assessing DNL in the same individual before and after menopause are challenging to conduct due to the duration of time required to follow up an individual as they complete the menopause transition. However, a cross-sectional study that compared premenopausal women to abdominal obesity-matched postmenopausal women showed no statistically significant difference in the rate of DNL (58). Despite compelling evidence in rodents, the impact of menopause on hepatic DNL in humans remains unclear.

Estrogen deficiency may impair hepatic fatty acid oxidation. E2 treatment increases the expression of carnitine palmitoyltransferase I (49), which catalyses the transfer of long-chain fatty acids into the mitochondrial matrix for the rate-limiting step of fatty acid oxidation (59). Furthermore, ovariectomy in rats reduces hepatic expression of peroxisome proliferator-activated receptor α, a master regulator of genes involved in fatty acid oxidation (60). E2 treatment of ovariectomized mice resulted in increased hepatic expression of uncoupling protein 2, a mitochondrial protein involved in dissipating metabolic energy and preventing oxidative stress (54). Furthermore, E2 treatment increased oxygen consumption in isolated hepatocytes undergoing the palmitate oxidation test, indicating improved long-chain fatty acid oxidation (54). Also, greater whole body dietary fatty acid oxidation is seen in women (menopausal status not specified) compared to age- and BMI-matched men with similar liver fat content (57). In a longitudinal study of perimenopausal women with annual indirect calorimetry testing for 4 years, whole-body fat oxidation decreased by 32% in women who became postmenopausal but was unchanged in women who remained premenopausal (61). However, not all studies in humans support an impact of menopause on fatty acid oxidation—no significant difference was found in the rate of fatty acid oxidation (assessed by measuring plasma levels of 3-hydroxybutyrate, a byproduct of fatty acid oxidation), between pre- and postmenopausal women matched for abdominal obesity (58).

Some evidence implicates estrogen in regulating hepatic fatty acid influx and lipid efflux. Gene expression of adipocyte differentiated related protein, a fatty acid uptake protein, is significantly upregulated in the liver of aromatase knockout mice, where its expression can be lowered with E2 replacement (62). Lipid export from the liver relies on the formation and secretion of VLDL-TG. Microsomal transport protein is responsible for incorporating TG in apoB100 in the first step of VLDL assembly. Estrogen may regulate this process, as expression of microsomal transport protein and VLDL-TG production is lower in ovariectomized rats (63). Interestingly, hepatic GPER activation mediates a reduction in proprotein convertase subtilisin/kexin type 9 mediated low-density lipoprotein receptor (LDLR) degradation (64). Therefore, estrogen deficiency may increase hepatic LDLR degradation. However, it is unclear how altered hepatic LDLR expression may impact metabolic phenotype, as hepatic LDLR has been shown to be both up- and downregulated in different studies on MASLD (65).

### Hepatic Glucose Metabolism

Estrogen regulates hepatic insulin signaling, hepatic insulin clearance, and pancreatic β-cell function. Estrogen deficiency leads to pathway-selective hepatic insulin resistance, where insulin is able to stimulate fatty acid synthesis but unable to suppress hepatic glucose production (66). Normally, insulin receptor tyrosine kinase activation in the liver leads to phosphorylation of insulin-receptor substrate (IRS) 1 and 2, which leads to activation of 1-phosphatidylinositol 3-kinase (PI3K) and subsequently Akt2. Akt2 inhibits the transcription of gluconeogenic genes phosphoenolpyruvate carboxykinase 1 and glucose-6-phosphatase by phosphorylation and nuclear exclusion of the transcription factor Forkhead box protein O1 (67). Estrogen deficiency disrupts hepatic insulin signaling by causing lipid-induced hepatic insulin resistance and impairing hepatocyte ERα signaling (24). As discussed in the previous section, hepatic intracellular lipid accumulation is a prominent feature of estrogen deficiency. Intracellular build-up of fatty acid metabolites, particularly diacylglycerol, triggers activation of protein kinase C ε (PKCε). While hepatic PKCε activation was previously proposed to mediate hepatic insulin resistance via phosphorylation of the insulin receptor in hepatocytes, the lack of protection against diet-induced insulin resistance in hepatocyte-specific PKCε knockout mice brings this hypothesis into question (68). Instead, the role of PKCε in mediating lipid-induced hepatic insulin resistance is thought to be related to altered adipose PKCε crosstalk with the liver (68). ERα activation can stimulate PI3K-Akt-Forkhead box protein O1 signaling to suppress hepatic glucose production, independent of IRS1/IRS2 phosphorylation (Fig. 1) (67). Indeed, estrogen replacement has been shown to correct defects in insulin sensitivity in an ovariectomy murine model but failed to correct whole-body and hepatic insulin resistance in mice with liver-specific ERα knockout after ovariectomy (8, 66). Increased hepatic glucose production contributes to the development of MASLD by driving hyperglycemia and consequent hyperinsulinemia, but hyperglycemia per se (independent of insulin) can also promote liver lipid accumulation by promoting the nuclear translocation of carbohydrate-responsive element binding protein (69).

Hepatic insulin clearance has a profound effect on serum insulin levels and glucose homeostasis, and decreased insulin clearance is implicated in the development of hyperinsulinemia in metabolic syndrome (70). One study has suggested that estrogen deficiency increases hepatic insulin clearance in mice (71). When compared to sham-operated mice, ovariectomized animals display increased hepatic expression of hepatic insulin-degrading enzyme (IDE) and a higher C-peptide/insulin ratio after a glucose load, consistent with increased hepatic insulin clearance. E2 replacement normalized IDE protein expression and C-peptide/insulin ratio (71). However, the role of IDE in hepatic insulin clearance is unclear, as liver-specific deletion of IDE does not cause hyperinsulinemia (70). Moreover, C- peptide/insulin ratios are poor estimates of hepatic insulin clearance due to varying kinetics of the C peptide compared to insulin (72). Hyperinsulinemic euglycemic clamp studies in humans suggest that whole-body insulin clearance may increase following estrogen treatment (73). Administration of IV conjugated estrogen (2.5 mg) to postmenopausal women (raising serum estrogen to levels seen in the midluteal phases premenopause) reduced serum insulin concentrations (73). Furthermore, increased insulin clearance was observed following 1-year of treatment with 35 µg ethinyl estradiol + 2 mg cyproterone acetate in healthy young women (74).

Estrogen deficiency also disturbs glucose homeostasis by interfering with pancreatic β-cell function. Estrogens regulate pancreatic β-cell insulin biosynthesis, survival, insulin secretion, and expression of GLUT2, the primary glucose sensor and transporter in rodent pancreatic cells (75, 76). Estrogen deficiency leads to pancreatic β-cell dysfunction, and E2 supplementation in rodent ovariectomy models restores pancreatic glucose-stimulated insulin secretion (71). In addition, E2 supplementation in rodents in vivo and in pancreatic islets in vitro limits β-cell apoptosis and sustains insulin production (77).

### Hepatic Amino Acid Metabolism

Dietary amino acids (AAs) regulate the transcriptional activity of ERα to orchestrate metabolic and reproductive functions (78). Analysis of liver transcriptomic and metabolomic profiles in response to short-term fasting or a high-fat diet revealed divergent responses in liver metabolism of male and female mice. In response to short-term fasting, male livers showed restrained gluconeogenesis and lipogenesis pathways, whereas female livers utilized AAs to generate substrates to fuel lipid synthesis (79). This effect does not seem to be a result of a whole-body metabolic shift, as these changes were not seen in adipose or skeletal muscle tissue. The liver-specific deletion of ERα resulted in the accumulation of AAs in female livers and a metabolic shift, contributing to liver lipid deposition (79). In female rodents fed a high-fat diet, AA homeostasis was preserved and no hepatic lipid accumulation was observed. However, in female hepatic ERα knockout mice fed a high-fat diet, disrupted liver AA homeostasis and significant liver lipid accumulation were observed (80). Given the ability of AAs to stimulate the activity of hepatic ERα, a number of studies have tested the metabolic impact of an AA-enriched diet on the liver. The AA-enriched diet was able to mitigate the transcriptomic changes, hepatic lipid deposition, and weight gain induced by ovariectomy in mice (81). This effect is mediated at least partly by hepatic ERα as the metabolic impact of the AA-enriched diet was blunted in liver-specific ERα knockout rodents (81). Targeting liver ERα with dietary interventions enriched with AA may therefore provide a strategy to limit metabolic derangements contributing to MASLD after menopause.

### Mitochondrial Dysfunction and Oxidative Stress

Hepatic mitochondrial dysfunction has a critical role in the development of MASLD by increasing oxidative stress, contributing to inflammation and impaired hepatic insulin resistance (4). Estrogen deficiency has been proposed to increase oxidative stress through the induction of mitochondrial dysfunction and reduced mitochondrial biogenesis. Both ovariectomized rats and rats fed a high-fat diet show increased markers of hepatic oxidative stress, which are improved after E2 treatment (82, 83). Estrogen appears to interact with peroxisome proliferator-activated receptor-γ coactivator-1 (PGC1), a family of transcriptional coactivators that enhance the activity of transcription factors via protein-protein interactions, to promote the expression of gene programs that enhance mitochondrial biogenesis and function (84). Studies have reported differing mechanisms for estrogen's antioxidant effects and involvement with PGC1A or PGC1B. Besse-Patin et al report that PGC1A promotes ERα expression while also acting as an ERα coactivator to mediate an antioxidant response involving increased expression of antioxidant genes such as superoxide dismutase 2 and glutathione peroxidase-1 (84). However, in HepG2 cells, E2 appears to signal via PGC-1B, as knockdown of PGC1B, and not PGC1A, impairs the E2-mediated effect on mitochondrial biosynthesis and function (82).

## Estrogen Deficiency Leads to Metabolic Inflexibility and Triggers Proinflammatory Signals From Adipose Tissue, Skeletal Muscle, and Gut Microbiome

**Figure 3.**
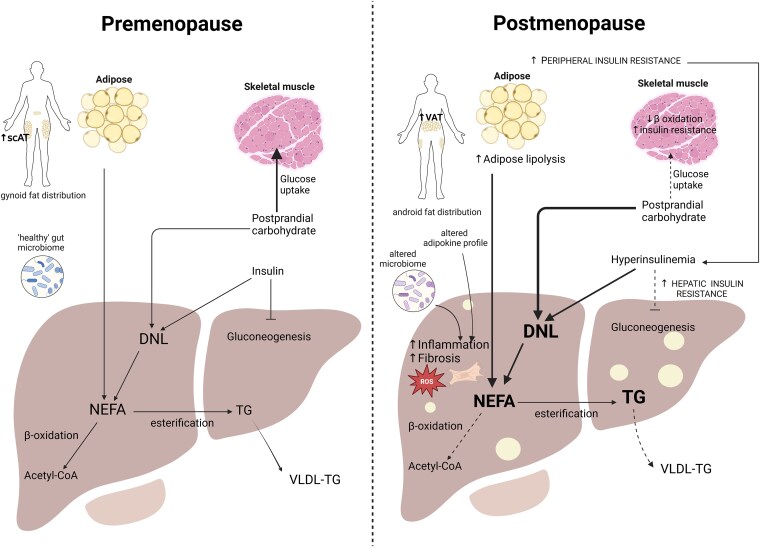
The effect of estrogen deficiency on liver, adipose, and skeletal muscle crosstalk. Left panel: An estrogen-replete (premenopausal) state. Adipose is mainly stored in subcutaneous adipose tissue depots, distributed in a gynoid pattern. The sources of non-esterified fatty acids (NEFAs) that contribute to liver triglyceride (TG) include de novo lipogenesis (DNL) using nonlipid precursors such as circulating carbohydrate, circulating NEFAs derived from adipose lipolysis, and dietary sources (not shown). NEFAs undergo β-oxidation to form acetyl-CoA or esterification to TG before being incorporated and secreted in very low density lipoprotein (VLDL). Insulin inhibits hepatic glucose production while stimulating DNL. After consumption of a meal, postprandial carbohydrate is taken up by skeletal muscle for glycogen synthesis. Right panel: An estrogen-deplete (postmenopausal) state. Adipose tissue is mainly stored in visceral adipose tissue depots, and increased adipose tissue lipolysis contributes to the pool of liver NEFA. Estrogen deficiency results in skeletal muscle and hepatic insulin resistance. The processes of β-oxidation and VLDL secretion are impaired, while DNL is upregulated by hyperinsulinemia and the diversion of postprandial carbohydrates. Multiple toxic insults converge to stimulate hepatic inflammation, and the altered adipokine secretion profile and microbiome contribute to hepatic inflammation. Estrogen deficiency leads to upregulation of fibrogenesis. Created in BioRender. Tomlinson, J. (2025) https://BioRender.com/5vqag23

Abbreviations: DNL, de novo lipogenesis; NEFA, nonesterified fatty acid; TG, triglycerides; VLDL, very low density lipoprotein.

Energy substrate utilization in muscle, liver, and adipose tissue is tightly regulated during fasting, feeding, and exercise states. Low insulin concentrations in the fasting state facilitate adipose tissue lipolysis to release NEFAs to fuel skeletal muscle activity and stimulate hepatic glucose production to maintain fasting plasma glucose levels (85). After a meal, higher levels of insulin stimulate uptake of glucose in skeletal muscle and liver while suppressing adipose tissue lipolysis. The estrogen-deficient state may disrupt this careful coordination of energy substrate uptake and utilization, impairing postprandial insulin-mediated suppression of adipose tissue lipolysis and leading to a chronic excess supply of circulating NEFA to the liver, eventually overwhelming its capacity for lipid removal to cause liver fat accumulation (86). This is a particularly important process in MASLD development, as almost two-thirds of fatty acids that contribute to hepatic triglyceride are derived from circulating NEFA (4). The impact of estrogen deficiency on the function of adipose and skeletal muscle is fundamental to the metabolic inflexibility that characterizes MASLD. Furthermore, estrogen deficiency alters the profile of circulating signals from adipose, skeletal muscle, and the gut microbiome to stimulate hepatic inflammation and MASLD development (Fig. 3).

### Adipose Distribution and Function

Estrogen deficiency promotes metabolic inflexibility of adipose tissue, adipose tissue redistribution, adipocyte inflammation, and altered adipokine secretion, which all drive the development of MASLD. In the transition to the postprandial (or fed) state, in response to insulin, adipose tissue lipolysis should be suppressed, lowering circulating NEFA levels and promoting a metabolic switch to glucose uptake and utilization (85). Estrogen plays an important role in the metabolic regulation of adipose tissue by enhancing insulin-mediated suppression of adipose tissue lipolysis and lowering circulating NEFA (86). An estrogen-deficient state is likely to impair this mechanism, inhibiting the postprandial insulin-mediated switch from fatty acid to glucose metabolism, leading to chronically elevated circulating NEFA. Estrogen replacement in postmenopausal women has been associated with a reduction in circulating NEFA during hyperinsulinemia (87), but the precise impact of HRT on adipose tissue insulin sensitivity has yet to be defined using gold-standard measurements of adipose tissue insulin resistance.

Menopause leads to a redistribution of adipose tissue toward central, visceral adipose depots (88). Premenopausal women possess a “gynoid” pattern of fat distribution where adipose is preferentially stored in subcutaneous and gluteofemoral depots, whereas postmenopausal women, like men, possess an “android” pattern of fat distribution where more adipose is preferentially stored centrally in the visceral depot (89). Subcutaneous and visceral adipocytes have distinct metabolic properties; visceral adipose accumulation is associated with increased risk of cardiometabolic disease, whereas subcutaneous adipose accumulation is considered metabolically protective (90). Findings in rodents and humans demonstrate that estrogen replacement, at least partially, reverses the adverse effect of the menopause on body fat distribution (19). The mechanisms underpinning estrogen's ability to regulate body fat distribution involve the regulation of depot-specific control of adipogenesis and adipose lipolysis. The reduced visceral adiposity seen in premenopausal females may be driven by ERα activation, which mediates a reduction in autophagy and adipogenesis within the visceral adipose depot via the mTOR-ULK1 pathway in female mice (91), and ERα deletion abolished sex-specific differences in autophagy and adiposity between male and female mice (91). In human adipocytes studied in vivo and in vitro, estrogen upregulates the expression of the antilipolytic α2A-adrenergic receptor in subcutaneous tissue but not visceral tissue, thereby maintaining gynoid fat distribution by preventing lipolysis in subcutaneous adipose depots (92). In vitro and preclinical rodent studies suggest that by inhibiting visceral adipogenesis and subcutaneous adipocyte lipolysis, estrogen appears to maintain a gynoid fat distribution in premenopausal women. The converse is seen in estrogen-deficient states; for example, in ovariectomized mice, basal and catecholamine-driven lipolysis in visceral adipocytes is impaired, while in subcutaneous adipocytes lipolysis was intact (93). Visceral adiposity is closely correlated with MASLD, and mechanistically this may be due to an excess flow of fatty acids and cytokines from visceral fat directly to the liver via the portal vein (94).

Altered adipokine secretion profile is likely to play a significant role in the development of MASLD following menopause. Adipokines affect wide-ranging processes implicated in MASLD development including hepatic lipid metabolism, inflammation, and fibrosis. Changes in adipokine secretion at menopause are likely to be linked with both lack of estrogen itself and the resulting body fat redistribution, as visceral fat predominance is linked with a more adverse adipokine secretion profile than gluteofemoral fat (90). Studying the effect of estrogen on the pattern of adipokine secretion has proved challenging. Although in vitro studies suggest that E2 treatment controls the secretion of adiponectin and leptin, results from animal models and observational human studies have not been consistent in the conclusions that have been drawn on the direction of adipokine change with estrogen deficiency (95). Adiponectin levels do not appear to vary with menopausal status (96). Estrogen correlated positively with leptin levels in premenopausal but not postmenopausal women (95). Estrogen deficiency may lead to changes in levels of omentin-1, resistin, and visfatin, but the impact of these changes on MASLD development is unclear (96).

### Skeletal Muscle Metabolism

Circulating NEFA is a major energy substrate for skeletal muscle, particularly during exercise, and estrogen's ability to promote skeletal muscle fatty acid oxidation may limit NEFA supply to the liver. Women demonstrate a lower exercise-induced respiratory exchange ratio than men, which indicates a higher ratio of fat oxidation to carbohydrate oxidation (97). This may be related to the positive effect of estrogen on the expression of major skeletal muscle enzymes involved in fatty acid uptake and oxidation by increasing peroxisome proliferation activator receptor α expression via ERα (98). Furthermore, muscle-specific ERα knockout mice demonstrate impaired fatty acid oxidation and lipid accumulation in muscle (99), and severe myosteatosis is significantly associated with MASH and fibrosis (OR 2-3) even after adjustment for confounders (100).

Skeletal muscle is the major tissue responsible for insulin-mediated glucose disposal, accounting for approximately 85% of all postprandial glucose uptake (101). Estrogen deficiency has a profound impact on insulin-stimulated glucose uptake in skeletal muscle; muscle-specific ERα knockout mice demonstrate a 45% reduction in insulin-stimulated glucose disposal during a hyperinsulinemia euglycemic clamp (99). Mechanistically, mitochondrial dysfunction plays a significant role in the development of skeletal muscle insulin resistance (101), and models of muscle-specific estrogen deficiency demonstrate aberrant mitochondrial morphology, increased production of reactive oxygen species, and impaired mitochondrial fission dynamics (99). Glucose is the main substrate for hepatic DNL, and elevated postprandial blood glucose promotes DNL, which may drive MASLD development. In vivo studies in humans have shown that skeletal muscle insulin resistance fuels hepatic lipid accumulation by causing the diversion of substrates from ingested carbohydrates away from skeletal muscle glycogen synthesis toward hepatic DNL (102, 103).

Estrogen deficiency has also been implicated in the development of sarcopenia, a condition of muscle tissue defined as loss of muscle mass, strength, and function. The prevalence of sarcopenia increases from the third to sixth decades of life but remains relatively constant afterwards and is higher in women ≥60 years compared to men of the same age (104). Sarcopenia may be important in the development of MASLD for 2 reasons. First, skeletal muscle is the major organ for fatty acid disposal, and lower skeletal muscle mass may therefore lead to higher levels of NEFA reaching the liver. Second, sarcopenia is believed to stimulate the secretion of a proinflammatory myokine profile, which aggravates liver inflammation (105). The relationship between sarcopenia and MASLD is likely to be bidirectional as the 2 entities share common pathogenic mechanisms such as insulin resistance and physical inactivity. On the one hand, sarcopenia is significantly associated with an increased risk of MASLD (106), and in patients with MASLD, sarcopenia is associated with an increased risk of liver fibrosis, independent of obesity or insulin resistance (107). In parallel, longitudinal studies have shown that patients with MASLD have accelerated skeletal muscle wasting compared to patients without MASLD (108). Whether estrogen deficiency has a causative role in promoting development of sarcopenia or whether the relationship between the estrogen deficiency and sarcopenia is a result of ageing is yet to be determined.

### Gut Microbial Dysbiosis

Gut microbial dysbiosis is recognized to play a major role in the development of MASLD. Gut microbiota regulate circulating estrogen concentration by modifying estrogen metabolism and secreting β-glucuronidase, which deconjugates estrogens into their active form (109). Lower estradiol concentrations are associated with lower gut microbial diversity and an altered profile of bacterial species (109). Compared to sham-operated mice, ovariectomized mice show lower expression of tight junction-associated proteins (indicating increased gut permeability) and harbor higher relative abundances of *Allobaculum* and lower relative abundances of *Bifidobacterium* and *Coprococcus* (109). To disentangle the metabolic impact of estrogen-deficiency driven gut microbial changes from the systemic effects of estrogen deficiency itself, fecal samples were transplanted from ovariectomized mice into male germ-free recipient gonadal-intact mice (109). The gnotobiotic mice receiving fecal microbiota transplantation from ovariectomized mice showed upregulation of hepatic proinflammatory genes, including the gene encoding glycoprotein hepatic fetuin A (109), which is elevated in patients with MASLD and is associated with MASLD severity (110). Therefore, there is emerging evidence to suggest that estrogen deficiency triggers a microbial dysbiosis that promotes metabolic dysfunction associated with MASLD.

## Anti-inflammatory and Antifibrotic Effects of Estrogen

The convergence of toxic stimuli such as insulin resistance, adipose tissue dysfunction, gut barrier dysfunction, lipotoxicity, hepatic endoplasmic reticulum stress, and mitochondrial dysfunction lead to a profound proinflammatory state that fuels the development of MASH. Hepatic inflammation is regulated by the balance between proinflammatory mediators and pathways that promote inflammation resolution, and estrogen deficiency is likely to interfere with this balance. Kupffer cells, triggered by the release of damage-associated molecular patterns from injured hepatocytes, propagate MASH inflammation by secreting proinflammatory cytokines IL1b, IL-6, and TNF-α to and activate HSCs by secreting TGFβ and platelet-derived growth factor. Activation of rat Kupffer cell ERα reduces secretion of cytokine-induced neutrophil chemoattractant 1 and neutrophil accumulation in response to hepatic injury (26). Kupffer cell polarization to proinflammatory M1 or repair M2 phenotypes is a key process determining the fates of continued inflammation or inflammation resolution. Activation of M2 macrophages derived from postmenopausal women is impaired (111). ERα activation inhibits hepatic macrophage M1 polarization, while ERα knockdown promoted hepatic steatosis, M1 macrophage infiltration, and fibrogenesis in female mice (112).

Upon activation, HSCs within the space of Disse transform into myofibroblast cells, which produce collagen/extracellular matrix. TGFβ-1 secreted from Kupffer cells and HSCs acts in a paracrine and autocrine manner and plays an important role in fibrogenesis by upregulating genes involved in the production of extracellular matrix. Pretreatment of rodent HSCs with estradiol before triggering HSC activation with hydrogen peroxide in vitro reduces TGFβ-1 expression and HSC activation (113). Mechanical cues such as a stiff surrounding extracellular matrix are also important for HSC activation. GPER1 activation promotes mechanical quiescence of HSCs of in vitro by increasing the activity of the small GTPase RhoA and reducing actomyosin contractility by inhibiting phosphorylation of myosin light chain 2 (32). ERβ activation has also been implicated in reducing hepatic fibrosis in vivo in a carbon tetrachloride rat model of cirrhosis; treatment with a selective ERβ antagonist (and not selective ERα or GPER1 antagonists) abolished the beneficial effect of E2 on liver fibrosis scores (29).

## Is There a Role for Menopausal HRT in the Prevention or Treatment of MASLD?

### Evidence From Clinical Studies on HRT and MASLD

HRT is an effective treatment for numerous issues related to menopause including vasomotor symptoms and osteoporosis and can lead to significant improvements in quality of life (114). In recent decades, the role of HRT has been heavily debated based on evolving evidence on the safety profile of the treatment, particularly in relation to risk of coronary heart disease and cancer. Initial results from early randomized placebo-controlled studies such as the Women's Health Initiative suggesting that HRT increased the risk of coronary heart disease and breast cancer across the board in postmenopausal women were inappropriately interpreted and communicated with the public, leading to sharp declines in HRT prescriptions (115, 116). Follow-up publications of the Women’s Health Initiative randomized controlled trials shed light on the more nuanced relationship between HRT and these outcomes (117). The clearer results of these more recent studies are driving a push for better awareness and education on the evidence base in relation to HRT prescribing internationally (116). Shifting public perceptions toward HRT are particularly evident in the UK, where the number of HRT prescriptions annually have increased dramatically in the past decade (14).

Unfortunately, our understanding of the hepatic impact of HRT has not paralleled the advancements in other organ systems, and the liver has so far largely been a bystander in the HRT debate. Despite the wealth of evidence implicating estrogen deficiency on wide-ranging processes driving multisystem metabolic dysregulation relevant to MASLD, there is a paucity of research on the effect of estrogen-based HRT on MASLD itself. Guideline-forming bodies such as the North American Menopause Society and the National Institute for Health and Care Excellence do not make recommendations on the impact of HRT on MASLD, and the North American Menopause Society highlights the need for further research on HRT and MASLD (118, 119). Existing clinical studies, summarized in Table 1, suffer from methodological limitations and have produced inconsistent results (120-129). Most of these studies report a beneficial effect of HRT on MASLD risk; some report a detrimental effect, while others do not report a significant result in either direction. A double-blinded placebo-controlled randomized study involving postmenopausal women with type 2 diabetes reported significant reductions in alanine aminotransferase (ALT) (−14 U/L, 95% CI −23 to −6 U/L, *P* = .002), aspartate aminotransferase (−9.2 U/L, 95% CI −14 to −5 U/L, *P* < .001), alkaline phosphatase (−60.8 U/L, 95% CI−80 to −42 U/L, *P* < .001), and gamma-glutamyl transferase (*P* = .035) following 6 months of oral 17β-estradiol (1 mg) and norethisterone acetate (0.5 mg) (121). An observational study of a cohort of 12 241 adults in the United States also used liver enzymes as a marker of MASLD and reported that MASLD (defined by elevated ALT, aspartate aminotransferase, or gamma-glutamyl transferase without evidence of an alternative explanation) was significantly less common in postmenopausal women using HRT compared to those not using HRT (OR 0.69; 95% CI 0.48-0.99; *P* < .05) (120). It is arguable that conclusions made on MASLD prevalence and severity in these studies are unreliable as liver enzymes have limited diagnostic utility and do not correlate well with histological severity of disease, particularly as ALT levels may decrease as liver fibrosis progresses to cirrhosis (130). In a nested case-control study using a UK primary care health record database that measured MASLD prevalence from clinical coding, there was a 54% elevated risk of MASLD in women without a history of HRT use (OR 1.54, 95% CI 1.33-1.77) but only a 15% elevated risk of MASLD in women who were using HRT (OR 1.15, 95% CI 1.02-1.30) (125). However, clinical coding is an unreliable marker of true MASLD status, particularly in a condition with very few clinical manifestations until advanced stages of the disease.

**Table 1.** Summary of published clinical studies on the effect of estrogen-based HRT on various characteristics of MASLD

Study design	Location, timeframe	MASLD criteria	Population	Form, dose, route, and duration of HRT	Comparator	Results	Reference
Cross-sectional	USA, 1988-1994	≥1 elevated liver enzyme level (ALT, AST, or GGT) and “no evidence of an alternative explanation”	Postmenopausal women, within a larger cohort of 12 241 adults	Details not specified	No reported use of HRT	MASLD was significantly less common in postmenopausal women using HRT compared to those not using HRT (OR 0.69; 95% CI 0.48-0.99; *P* < .05)	(120)
Double-blind, placebo controlled randomized study (*post hoc* analysis)	UK, 1998-2000	Serum liver enzymes (ALT, AST, ALP, GGT)	Clinically and biochemically postmenopausal (1 year since last menses and FSH > 20 IU/L) with T2DM	Oral 1 mg 17β-estradiol and 0.5 mg norethisterone acetate for 6 months (n = 19)	Placebo (n = 23)	In those using HRT, there were significant reductions in ALT (−14 U/L, 95% CI −23 to −6 U/L, *P* = .002), AST (−9.2 U/L, 95% CI −14 to −5 U/L, *P* < .001), ALP (−60.8 U/L, 95% CI −80 to −42 U/L, *P* < .001), and GGT (*P* = .035).	(121)
Cross-sectional	Brazil, 2009-2011	Presence of steatosis on abdominal ultrasound, alcohol consumption < 20 g/day and absence of other liver diseases	Absence of menstruation for 12 consecutive months in healthy women. FSH > 50 mIU/mL to confirm menopausal status in women with history of hysterectomy without oophorectomy	Estrogen in participants with history of hysterectomy, estrogen and progesterone in participants with uterus intact, or tibolone. Oral or transdermal route for more than 6 months (total n = 53)	No reported use of HRT (n = 198)	Prevalence of MASLD was lower in the group who used HRT (14/53, 26.4%) compared to the group who denied using HRT (79/198, 39.9%). Postmenopausal women with MASLD using HRT had lower levels of GGT (*P* = .001), ALT (*P* < .01), ferritin (*P* < .001), and HOMA-IR (*P* < .001) than postmenopausal women who did not.	(122)
Cross-sectional	Korea, 2003	“Fatty liver” on ultrasound, with no evidence of viral hepatitis and consumption <20 g alcohol/day (sex-specific)	An analysis of 2644 women, including some menopausal women	“Estrogen medication” (n = 167) Not specified if this term includes contraceptive use in premenopausal women	—	The association between estrogen medication and risk of MASLD in women was assessed within a multivariate analysis. History of “estrogen medication” in all women was significantly associated with increased MASLD risk (OR 2.12, 95% CI 1.27-3.56, *P* < .05). However this may be a reflection of the association between MASLD and menopausal status (OR 1.71, 95% CI 1.27-2.32, *P* < .001)	(123)
Prospective cohort	Japan, 2001-2003	MASLD diagnosed on abdominal ultrasound	Postmenopausal women, defined by cessation of menses for 1 year	Details not specified	—	A logistic regression analysis was performed in a cross-sectional analysis at baseline to assess the relationship between HRT and MASLD. After adjusting for confounding factors, the adjusted OR for an association between HRT and MASLD was not statistically significant after adjusting for age and metabolic syndrome (adjusted OR 1.55, 95% CI 0.92-2.61, *P* = .096) The individuals who did not have MASLD were followed up for an average duration of 414 days, and a logistic regression analysis was performed, which found that HRT was not a statistically significant risk factor for MASLD (adjusted OR 1.32, 95% CI 0.6-2.88, *P* = .49)	(124)
Nested case-control	UK, 1988-2017	Coding of MASLD within CPRD database	Female controls (n = 40 344) matched to cases of MASLD (n = 10 082 cases)	Estrogen only or estrogen-progesterone combination	—	Oophorectomy and HRT were both separately associated with a >50% increased risk of MASLD compared to women without oophorectomy or HRT use. The positive association between HRT and MASLD may be at least partially due to the menopausal status of women using HRT. Using an analysis of the interaction between oophorectomy and HRT use, there was evidence of a negative interaction from HRT use on the relationship between oophorectomy and MASLD. Oophorectomy was associated with a 54% elevated risk of MASLD (OR 1.54, 95% CI 1.33-1.77) in women with no HRT use but only a 15% elevated risk of MASLD (OR 1.15, 95% CI 1.02-1.30) among women with HRT use.	(125)
Cross-sectional	USA, 2007-2010	Histological diagnosis of MASH	Postmenopausal women (n = 199)	“Estrogen replacement therapy”—likely various regimens	—	Exploratory analysis and after adjusting for confounders, HRT was associated with a 50% risk reduction in fibrosis although this was not statistically significant.	(126)
Cross-sectional study, data from 3 different clinical studies	USA	Histological diagnosis of MASLD	Postmenopausal women (n = 489)	Self-reported HRT use	No HRT	HRT was associated with more severe hepatic lobular inflammation in postmenopausal women (*P* < .05). Additional analysis concluded that progesterone use, rather than estrogen, was responsible for the increased histological severity of disease.	(127)
Nested case control	USA, cohort enrollment 1993-1996 (multiethnic groups)	MASLD cases by Medicare claims data (ICD-9 coding)	Postmenopausal women; status determined by participant self-reported menstruation information	Estrogen-only HRT (MASLD cases = 453, controls = 3612) Estrogen and progesterone HRT (MASLD cases = 381, controls 3556)	No HRT	Compared to postmenopausal women without a history of using HRT, the risk of MASLD was higher in postmenopausal women who had a history of using estrogen-only HRT (OR = 1.18, 95%CI = 1.00-1.38) or estrogen and progesterone HRT (OR = 1.25, 95% CI = 1.07-1.46)	(128)
Retrospective cohort study	Korea, 2016-2020	Hepatic steatosis on abdominal ultrasound	Amenorrhea ≥ 12 months or serum FSH ≥ 20 IU/L	Low (≤ 1 mg) or high (2 mg) 17β-estradiol, low (<0.625 mg) or high (0.625 mg) conjugated estrogen administered orally Natural vs standard progestogens 12-month duration of treatment	Transdermal estrogen used at equivalent dose of oral	The prevalence of MASLD in the transdermal group decreased from 24% to 17.3% after 12 months of treatment but increased from 25.3% to 29.4% in group receiving oral HRT. No changes in the prevalence of MASLD were found between groups receiving low vs high doses of estrogen, and no differences were found between groups receiving natural vs standard progestogens.	(129)

Abbreviations: ALP, alkaline phosphatase; ALT, alanine aminotransferase; AST, aspartate aminotransferase; CI, confidence interval; CPRD, Clinical Practice Research Datalink; GGT, gamma-glutamyl transferase; HOMA-IR, homeostatic model assessment of insulin resistance; HRT, hormone replacement therapy; ICD-9, International Classification of Diseases, Ninth Revision; MASH, metabolic dysfunction-associated steatohepatitis; MASLD, metabolic dysfunction-associated steatotic liver disease; NAFLD, nonalcoholic fatty liver disease; NASH, nonalcoholic steatohepatitis; OR, odds ratio; T2DM, type 2 diabetes mellitus.

The use of abdominal ultrasound in the detection of steatosis is an established methodology, and studies on HRT that employ abdominal ultrasound are likely to detect MASLD more reliably in their populations (131). However, ultrasound remains a relatively insensitive method for detecting lower levels of hepatic steatosis. The 4 studies in Table 1 that used abdominal ultrasound in their workup for MASLD reported conflicting conclusions (122-124, 129). One study assessed the association between HRT and MASLD without considering or adjusting for the underlying menopausal status of participants (123). This cross-sectional study conducted in Korea reported a positive association between “estrogen medication” and MASLD risk (OR 2.12, 95% CI 1.27-3.56, *P* < .05), though this relationship is likely to be confounded by the association between estrogen “medication” and menopausal status (123). A retrospective cohort study, also conducted in Korea, assessed the impact of route and dose of HRT administration on MASLD risk (129). The prevalence of MASLD in the group receiving transdermal estrogen decreased from 24% to 17.3% after 12 months of treatment but increased from 25.3% to 29.4% in group receiving oral estrogen (129). As the liver receives a larger bolus of HRT from the orally administered route compared to the transdermal route and different routes of administration lead to varying effects on circulating lipid profile, oral and transdermal HRT are likely to have distinct impacts on MASLD development (49). Furthermore, oral and transdermal HRT confer divergent effects on substrate oxidation. In a study of 23 postmenopausal women with a history of hysterectomy, after 12 months of daily oral conjugated estrogen (0.625 mg) or daily 17β-estradiol transdermal patches (50 μg), carbohydrate oxidation increased in the oral group and decreased in the transdermal group, whereas fatty acid oxidation decreased in the oral group and increased in the transdermal group (132). Increased fat oxidation may enhance insulin sensitivity (133), counteracting the pathological processes implicated in MASLD; however, this specific study did not measure liver fat content (132). The results of observational studies are limited by selection and recall bias (129). Most notably, these studies are limited by healthy user bias where women who use HRT have systematically different characteristics (eg, socioeconomic status) than those who do not, which may be sources of residual confounding and therefore may influence some of the seemingly beneficial reported outcomes of HRT (134). Prospective interventional studies measuring liver fat content are required to validate the impact of administration route on MASLD development.

A cross-sectional study incorporating data from 3 separate clinical studies of patients with histologically confirmed MASLD reported that HRT was associated with more severe hepatic lobular inflammation in postmenopausal women (*P* < .05) (127). This concerning result was followed up with a subgroup analysis that led the authors to conclude that progesterone use, and not estrogen, was responsible for the increased histological severity of disease (127). Research on the impact of progesterone supplementation on the liver is very limited, but the results of 1 preclinical study support a detrimental effect on liver lipid metabolism. In this study, progesterone upregulated genes involved in DNL in Hep3B cells in vitro via progesterone receptor B activation and in ovariectomized mice fed a high-fat diet, progesterone increased serum triglycerides and led to liver lipid accumulation (135). Progestogens are believed to drive some of the established risks of HRT (eg, breast cancer), and while different classes of progestogens possess distinct metabolic activities (136), existing studies on HRT and MASLD that consider progestogens only do so by studying the effect of progestogens as a single group (127), thus limiting their interpretation. However, estrogen-only HRT is not free from implications in worsening MASLD. A nested case control study, where people with MASLD were identified using Medicare claims data and matched to controls without liver disease by birth year, ethnicity, and Medicare enrollment duration, reported that both estrogen-only HRT (OR 1.18, 95% CI 1.00-1.38) and combined estrogen and progesterone HRT (OR 1.25, 95% CI 1.07-1.46) were associated with a higher risk of MASLD compared to postmenopausal women with no history of HRT use (128). Although it seems confusing that HRT could adversely affect a condition driven by estrogen deficiency, this clinical data should be carefully considered and further explored. Possible explanations for how HRT could adversely affect MASLD development include adverse effects from the progesterone component of HRT (which is infrequently considered in existing studies), nonphysiologically high hepatic estrogen exposure from oral estrogens, and the inability to replicate the natural oscillatory patterns of hepatic estrogen exposure in the premenopausal state (10). The validity of many of these existing studies is limited by either their small sample sizes, study design (often observational or exploratory analyses of studies designed for a separate purpose), and the use of inaccurate methods to ascertain MASLD diagnosis or progression. Future translational studies should adopt an interventional design and carefully consider the impact of estrogen dose, route of administration, duration of treatment, and impact of progesterone.

An individualized approach is needed for the commencement and continuation of HRT, and discussions between healthcare practitioners and each woman should take into account their personal benefit vs risk profile to aid shared decision-making. Given the high burden of MASLD in women and the poorer outcomes from the disease compared to men, it is a priority to clarify the impact of HRT on MASLD development and severity to give women the opportunity to make more informed decisions, potentially leading to improved health outcomes after menopause.

### Evidence From Clinical Studies on the Effect of HRT on Extrahepatic Pathways Involved in MASLD

While the evidence from “liver-centric” clinical studies assessing the impact of HRT on MASLD is limited, we can gather insights into this process by inferring from studies exploring the effect of HRT on relevant extra-hepatic pathways or outcomes relevant to MASLD.

Given the role of the liver as a center for lipid uptake, production, and secretion, studies that assess the impact of HRT on circulating lipid profiles may shed light on processes relevant to MASLD development. As mentioned earlier, oral and transdermal HRT lead to divergent effects on circulating lipid profiles (49). Oral estrogen increases production of serum VLDL and high-density lipoprotein cholesterol and decreases low-density lipoprotein cholesterol (49). Transdermal estrogen does not appear to increase VLDL-TG concentrations and has a milder effect on increasing high-density lipoprotein cholesterol and lowering low-density liooprotein cholesterol (49). This discrepancy has been attributed to the effect of first-pass metabolism on orally administered estrogen, but the exact mechanism for this, and the impact on total liver fat content, is not clear. Stable isotope tracer experiments have confirmed that transdermal estradiol does not affect VLDL-TG concentration by altering VLDL-TG secretion and that instead it achieves this effect by increasing VLDL-TG plasma clearance (137). While the increased liver VLDL secretion seen following oral HRT is fundamentally a process that removes lipid from the liver, increased VLDL secretion may simply be a product of increased liver lipid content, and rates of triglyceride synthesis can exceed VLDL-TG secretion to cause steatosis (138). Metabolomic data from a placebo-controlled randomized controlled trial on oral conjugated estrogens (0.45 mg/day) and bazedoxifene (20 mg/day) (CE/BZA) in postmenopausal women sheds light on the changes in pathways in hepatic lipid accumulation with HRT (139). Daily treatment with CE/BZA increased serum diacylglycerol and triacylglycerol species without changes in these lipids in skeletal muscle, suggesting a hepatic origin of these lipids from increased esterification and export of fatty acids into VLDLs (139). CE/BZA also reduced levels of long-chain acylcarnitines compared to the placebo-treated group, which may suggest decreased β-oxidation (139). Although this study reveals changes in processes that govern liver lipid content, without a corresponding measurement of liver fat content and ascertainment of the relative change and balance between each of these processes, limited further conclusions about the impact of CE/BZA on MASLD development can be made.

**Figure 4.**
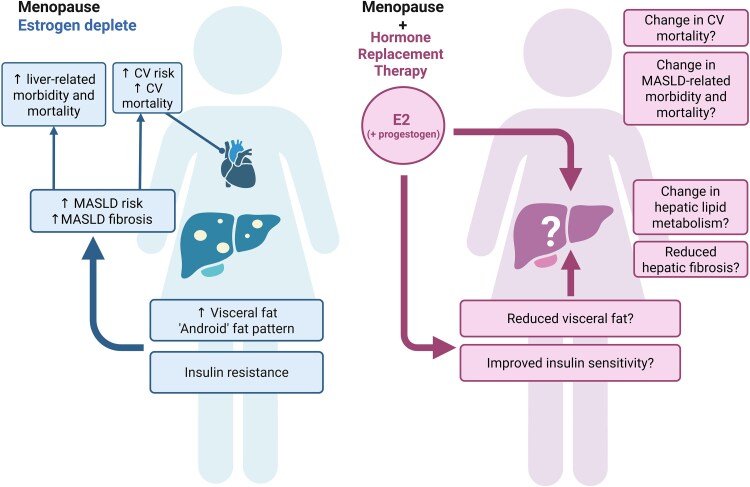
Potential effects of Hormone Replacement Therapy (HRT) on the multisystem features of metabolic dysfunction-associated steatotic liver disease (MASLD). The precise impact of HRT on the multisystem features of MASLD in humans is unclear, and existing studies are observational or post hoc analyses. Although some studies have demonstrated beneficial effects from HRT (121, 122), others have demonstrated a harmful effect (which may be related to the progestogen) (127), and guidelines recommend caution when prescribing HRT to those with pre-existing liver disease (142). Transdermal estrogen may have more beneficial effects on MASLD development than oral estrogen (129) and carries a lower risk of venous thromboembolism, cholestasis, and stroke (118, 143). In order to establish the precise effect of HRT on MASLD development, well-designed prospective studies that use accurate methods to measure MASLD development are needed. Created in BioRender. Tomlinson, J. (2025) https://BioRender.com/vqcwsaz

Abbreviations: HRT, hormone replacement therapy; MASLD, metabolic dysfunction-associated steatotic liver disease.

Insulin resistance has a pivotal role in the development of MASLD, leading to metabolic inflexibility and causing chronically elevated circulating NEFA concentrations (4). Despite the evidence from preclinical studies that emphasize the role of estrogen deficiency in the development of insulin resistance (86, 99), the impact of HRT on insulin sensitivity in postmenopausal women is unclear. A meta-analysis on the effect of HRT on components of metabolic syndrome concluded that HRT significantly improved homeostatic model assessment of insulin resistance (−35.8%, CI −51.7 to −19.8%) (140). However, results from hyperinsulinemic euglycemic clamp studies in postmenopausal women using HRT, which provide gold-standard measurements of insulin sensitivity, have been conflicting, and some studies have reported a worsening of insulin resistance in women using estrogen-only or combined estrogen and progesterone HRT (141). This discrepancy may be related to HRT regimen, route of administration, and timing of initiating HRT relative to menopause (101). HRT may also help to alleviate the increase in visceral adiposity associated with menopause to some extent (119). The potential impacts of HRT on MASLD are summarized in Fig. 4.

### Potential Risks of HRT in People With Liver Disease

Besides the well-documented risks of HRT in the general population (115, 118), there are some notable areas of concern in people with preexisting liver disease. First, venous thromboembolism is common in individuals with cirrhosis (144), and oral estrogen is prothrombotic (118). Furthermore, HRT promotes cholestasis (119), and dysregulated bile acid homeostasis may promote lipid accumulation and lipotoxicity, driving MASLD (145). In the advanced stages of liver disease, when hepatic metabolism and drug clearance are affected, safety is a concern, and plasma levels of estrogen can be elevated (146). Whilst some studies have reported that HRT is associated with a lower risk of liver cancer in women (147), the exact relationship between HRT and liver cancer remains to be elucidated. Some early studies had implicated oral contraceptives in the progression of hepatic adenomas (148), and current guidelines advise the avoidance of estrogens in patients with adenomas (149). Notably, earlier preparations of oral contraceptive pills contained higher doses of estrogen compared to contemporary preparations (and certainly less than contemporary HRT regimens) (150) and were studied in the context of predominantly estrogen-replete populations. Perhaps for these reasons, some guidelines suggest that liver disease is a caution (142) or even a contraindication (119) for the use of HRT. Although there may be safety concerns in those with more advanced liver disease, HRT is likely to be a more viable option in those with early-stage disease. Given the prevalence of MASLD, it is unlikely that these guidelines were written with the intention of excluding all women with MASLD from HRT, and real-world data suggests that many women with MASLD are using HRT (125). Transdermal estrogen does not increase the risk of venous thromboembolism (118), has a lower risk of cholestasis than the oral route (143), and may be a more amenable form of HRT for people with MASLD, which is supported by the results of 1 observational study (129). Many individuals with MASLD will have cardiovascular risk factors, and these should be considered carefully as part of a shared discussion on initiation and continuation of HRT, especially where nonoral estrogen therapy is also considered as a lower risk option (151).

## Other Treatment Strategies for MASLD in Postmenopausal Women

### Lifestyle and Dietary Interventions

Even if further studies prove that HRT is an effective treatment strategy for MASLD in postmenopausal women, HRT will not be a universally suitable treatment for all, as the benefit vs risk profile of HRT varies between individuals. Furthermore, the duration of HRT use is carefully considered, as risks including breast cancer and cardiovascular disease increase with duration of HRT use (152). Research on other menopause-specific treatment strategies for MASLD is also warranted. Lifestyle interventions, including weight loss and exercise, are a mainstay of MASLD management in women (153). Despite promising data from estrogen-deficient rodent models on the impact of AA supplementation (81), the impact of AA-based dietary interventions is yet to be tested on MASLD in postmenopausal women. In men, the impact of AA supplementation seems to vary, dependent upon the composition of the AA profile (154). In women with obesity and polycystic ovary syndrome, dietary essential AA supplementation (histidine, isoleucine, leucine, lysine, phenylalanine, threonine, and valine) reduced hepatic fat fraction, liver enzymes, plasma triglycerides, and plasma VLDL (155).

### Targeted Estrogen Receptor Therapies

Hepatic ERα is a potential target for pharmacological interventions given its significant role in controlling hepatic metabolism. As expected from our understanding of the function of hepatic ERα, administration of a specific ERα agonist (16α-LE2 3  μg/kg/day) alleviated hepatic steatosis in aromatase knockout mice (156). However, Erα-targeted therapies are likely to worsen outcomes in carriers of the highly common *PNPLA3* p.I148M variant, where ERα activation may increase the number of pathogenic malfunctioning defective *PNPLA3* enzymes on lipid droplets to drive liver lipid accumulation and worsen MASLD severity (47). Utilizing genetic variants to personalize therapies will avoid this potential issue, and stratifying women based on risk of MASLD progression at menopause will identify those who would most benefit from pharmacological treatment. The beneficial effects of ERα agonism in dampening hepatic proinflammatory activity of Kupffer cells and ERβ agonism in limiting fibrogenesis suggest that targeted ER therapies might be a means to limit hepatic inflammation in MASH and prevent worsening of fibrosis. The ERβ agonist LGND2 reduces hepatic infiltration of inflammatory cells and collagen deposits in C57BL/6 mice fed a high-fat diet (157). Selective GPER1 agonism is another potential treatment strategy. In an ovariectomized rodent model, administration of the specific GPER1 agonist G1 protects against obesity, hepatic steatosis, inflammation, and fibrosis through AMPK-mediated signaling (11). These targeted ER therapies are all currently experimental, although GPER1 agonists have recently entered clinical trials for the treatment of refractory solid organ malignancies where they appear to be well tolerated (NCT04130516). Further investigation of targeted ER agonist therapy is warranted to establish their safety profile and to determine if there are any advantages over nonspecific ER activation achieved by HRT.

### Targeting Downstream Effectors of ER Activation

While ER-targeted therapies are likely to suffer from some of the same risks as estrogen supplementation (115), targeting selected downstream effectors of ER may represent an opportunity to capture the metabolic benefits while bypassing adverse effects associated with estrogens. TEA domain transcription factor 1 (*TEAD1*) expression is suppressed by systemic ER stimulation in male mice fed a high-fat diet, and *TEAD1* expression is upregulated in people with MASLD (158). In a primary human hepatocyte spheroid model, *TEAD1* inhibition reduced hepatic steatosis by altering central metabolic pathways controlling lipid homeostasis (158). Regulation of appropriate pathways downstream of the ER could also be an effective therapeutic strategy regardless of sex.

### Estrogen Metabolites

As the liver is the primary site for estrogen metabolism, some actions attributed to E2 in studies may be the result of the formation of biologically active E2 metabolites. 2-hydroxyestradiol and 2-methoxyestradiol, the respective products of CYP450 and catechol-O-methyl-transferase from E2 metabolism, have demonstrated increased potency compared to E2 for inhibitory activity of HSCs. Concentrations of E2 in the range of ≥10^−7^ mol/L inhibited HSC proliferation and extracellular matrix (ECM) secretion, whereas the same magnitude of effects was seen at concentrations of 2-methoxyestradiol in the range of 10^−9^ mol/L (159). Therefore, leveraging the activity of E2 metabolites may potentially be an effective therapeutic strategy for reduction of fibrosis in MASLD.

### Phytoestrogen Therapy

Phytoestrogens are another class of compounds that have been implicated as potential treatments for MASLD. These plant-derived compounds possess a similar structure to human estrogens and bind to estrogen receptors to regulate wide-ranging metabolic processes implicated in MASLD (160). Genistein, a soybean derivate and the most studied phytoestrogen, reduces hepatic triglyceride and serum NEFA in rodent models of MASLD by various mechanisms including inhibition of hepatic DNL and stimulation of hepatic fatty acid oxidation (160). In a diet-induced rat model of MASH, genistein supplementation reduced multiple markers of severity of liver inflammation including hepatic TNF-α level, inflammatory cell inflammation, and overall histological severity (161). While there are promising results in rodent models, clinical studies on phytoestrogens in humans are currently limited. A double-blinded randomized clinical trial involving 10 female and 31 male adults (mean age 43.6 years) with MASLD assessing the impact of daily 250 mg genistein supplementation for 8 weeks reported reduced serum levels of insulin (*P* = .001), homeostasis model assessment for insulin resistance (*P* = .041), serum IL-6 (*P* = .018), serum TNF-α (*P* = .045), serum malondialdehyde (*P* = .004), and waist to hip ratio (*P* = .021) with genistein compared to placebo (162). Another placebo-controlled randomized controlled trial assessing the impact of daily 54 mg genistein supplementation on 54 postmenopausal women concluded that genistein significantly reduced markers of insulin sensitivity and systemic inflammation (163). Despite promising impacts on surrogate metabolic indices, genistein remains an experimental treatment, and further studies on phytoestrogens should clarify the efficacy of these treatments using liver-centric outcomes to determine MASLD severity.

## Conclusions and Future Directions

There is now compelling evidence from epidemiological and preclinical studies demonstrating that estrogen deficiency promotes the development and progression of MASLD through several processes. Phytoestrogens and targeted ER agonists are experimental treatments with unknown safety profiles and uncertain clinical efficacy and are currently very far from achieving marketing authorization and reaching clinical practice. HRT is already widely available for other indications in menopause and may be an effective treatment strategy for MASLD; however, existing studies on the impact of HRT on MASLD are burdened heavily by methodological limitations and have yielded inconsistent findings. Additionally, there is a lack of studies that have explored the impact of specific progestogens on MASLD development and progression. Well-designed, translational studies on HRT that use accurate, noninvasive markers of MASLD severity are needed. High-quality clinical data from such studies will inform the development of clinical practice guidelines on HRT and MASLD, to guide counseling and informed decision-making for postmenopausal women and determine whether HRT has potential as a disease-modifying treatment for MASLD. As the poor outcomes of MASLD in women have become clearer and the uptake of menopausal HRT begins to increase, the question of how HRT affects MASLD development in menopausal women cannot be overlooked any further. There is a case to be made that HRT should be a focus of the search for sex-specific MASLD treatment strategies.
